# Cooperative Nuclear Localization Sequences Lend a Novel Role to the N-Terminal Region of MSH6

**DOI:** 10.1371/journal.pone.0017907

**Published:** 2011-03-17

**Authors:** Natalie R. Gassman, Jill E. Clodfelter, Anita K. McCauley, Keith Bonin, Freddie R. Salsbury, Karin D. Scarpinato

**Affiliations:** 1 Department of Cancer Biology, Wake Forest University School of Medicine, Winston Salem, North Carolina, United States of America; 2 Department of Biology, Wake Forest University, Winston Salem, North Carolina, United States of America; 3 Department of Physics, Wake Forest University, Winston Salem, North Carolina, United States of America; 4 Comprehensive Cancer Center, Wake Forest University School of Medicine, Winston Salem, North Carolina, United States of America; University of Massachusetts Medical School, United States of America

## Abstract

Human mismatch repair proteins MSH2-MSH6 play an essential role in maintaining genetic stability and preventing disease. While protein functions have been extensively studied, the substantial amino-terminal region (NTR*) of MSH6 that is unique to eukaryotic proteins, has mostly evaded functional characterization. We demonstrate that a cluster of three nuclear localization signals (NLS) in the NTR direct nuclear import. Individual NLSs are capable of partially directing cytoplasmic protein into the nucleus; however only cooperative effects between all three NLSs efficiently transport MSH6 into the nucleus. In striking contrast to yeast and previous assumptions on required heterodimerization, human MSH6 does not determine localization of its heterodimeric partner, MSH2. A cancer-derived mutation localized between two of the three NLS significantly decreases nuclear localization of MSH6, suggesting altered protein localization can contribute to carcinogenesis. These results clarify the pending speculations on the functional role of the NTR in human MSH6 and identify a novel, cooperative nuclear localization signal.

## Introduction

The mismatch repair system (MMR) is one of the key DNA repair systems, ubiquitously found in most organisms investigated to-date. It recognizes and repairs errors occurring during DNA replication, increasing the fidelity of this essential cellular process [1], [2]. Defects in these proteins dramatically increase genetic instability, providing the underlying cause for all “hallmarks of cancer” [3].

Best studied in *Escherichia coli*, a homodimer of MutS proteins recognizes mismatched DNA and initiates repair. In mammalian cells, MMR shows higher complexity. The homodimeric MutS is replaced by two heterodimeric complexes consisting of MSH2-MSH6 and MSH2-MSH3 with different, but overlapping substrate specificity [4], [5], [6]. Binding of these heterodimeric complexes to mismatched DNA initiates subsequent, consecutive steps that involve ATP binding and hydrolysis to recruit downstream proteins and lead to the excision of the mismatched DNA and resynthesis.

**Figure 1:**
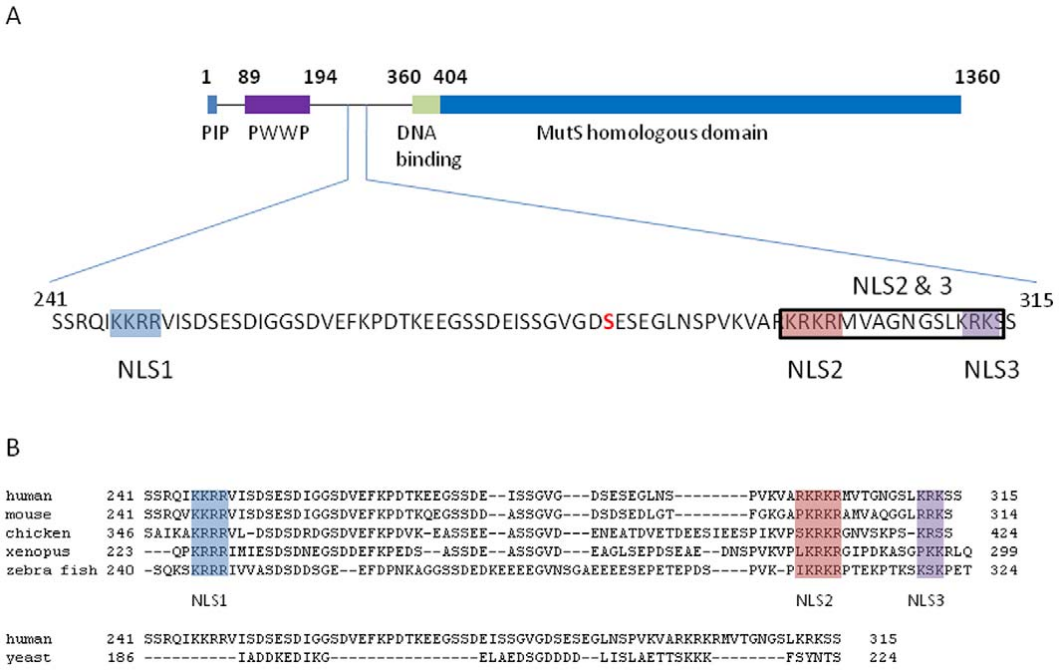
Identification of three nuclear localization sequences in an uncharacterized region of the N-terminal region of MSH6 that are only conserved in higher eukaryotes. (A) Schematic drawing of human MSH6 detailing its N-terminal region. PCNA interaction motif (PIP) is shown in blue, PWWP domain in purple, and predicted non-specific DNA interaction domain is shown in green. Identified potential nuclear localization sequences are highlighted in blue, red and purple (NLS1, NLS2, and NLS3). Cancer mutation S285I is indicated in bold. (B) Clustal alignment of MSH6 N-terminal regions from different eukaryotic species demonstrating location of the NLSs. Yeast Msh6 contains no comparable region to those found in higher eukaryotes, as shown. The higher eukaryotic species contain highly similar and closely spaced NLSs to those found in human MSH6.

Additional complexity is added to the mammalian proteins by the addition of an extra 300–400 amino acid stretch to the N-terminus of MSH6 and MSH3 proteins, not found in their prokaryotic counterparts or in MSH2 (Figure 1A). This N-terminal region (NTR) of the protein has attracted significant attention in the scientific world, primarily due to the fact that despite its size, few signature motifs are identifiable. The most extensively studied function for this NTR resides in its far N-terminus which contains a functional PCNA binding motif (Figure 1A, “PIP”) [7],[8] that tethers the MMR machinery to the replication fork machinery [9]. Subsequently, small-angle X-ray scattering described the NTR of yeast Msh6 as an unstructured tether to PCNA, providing a flexible linker between the protein and PCNA [10]. In human cells, such an unstructured tether could not be identified by the same technique, suggesting a difference between yeast and human protein [11]. The only other functional consequence of mutations within the NTR identified non-specific DNA binding that involves amino acids 228–299 of yeast Msh6 [8] and a PWWP domain in the human MSH6 [12] (Figure 1A). Despite these earlier reports, the function of remaining parts of the human MSH6-NTR remains enigmatic.

Much effort has been spent on describing the functions of MMR proteins, culminating in detailed insight into their mechanism, yet how these proteins are localized in cells and the determinants for this localization are not well defined. Only recently has one publication attempted to address the nuclear and cytoplasmic localization for the yeast MSH proteins [13]. This study found that yeast Msh6 was excluded from the nucleus in the absence of Msh2, and that, while Msh2 could localize to the nucleus independent of Msh6, its protein levels were diminished in the absence of its partner protein [13]. This suggests that transport and stability of yeast Msh2 and Msh6 are synergistically enhanced by heterodimerization; a phenomenon that has been observed for nuclear import of human MLH1 and PMS2 as well [13], [14], [15].

Though yeast model systems are frequently used as an equivalent for the human system, recent findings suggest discrepancies between lower and higher eukaryotic systems. Whether above findings therefore hold true for human MSH2 and MSH6 remains to be determined; the only information on subcellular localization for the human proteins was indirectly found after exposure to DNA damaging agents. In these studies, MSH2 and MSH6 were found to re-localize to sites of damage after exogenous insult [16], [17], [18], [19]. Deletion of the NTR from MSH6 was also found to affect accumulation of MSH6 to sites of DNA damage independent of PCNA binding [19].

Classical nuclear localization signals have been divided into monopartite, containing a single cluster of basic amino acid residues, and bipartite sequences, containing two clusters of basic amino acid residues separated by 10–12 amino acids. Loose consensus sequences have been established for both monopartite, K(K/R)*X*(K/R), and bipartite signals, (K/R)(K/R)X_10–12_(K/R)_3–5_, where (K/R)_3–5_ represents at least three either lysine or arginine out of the five consecutive amino acids [20]. Examination of the human MSH2 and MSH6 amino acid sequences identified no predicted NLSs in MSH2 [15], [21], [22] and four predicted NLSs in MSH6, all residing in the NTR region [22].

Utilizing confocal microscopy and site-directed mutagenesis, we demonstrate that three functional monopartite NLSs are located in a region of unknown function in the human MSH6 NTR. Furthermore, we show that, while these individual NLSs have varying degrees of activity, they show unusual cooperativity in localizing MSH6 to the nucleus. Absence of this NTR region or deletions of individual NLSs affects nuclear transport of MSH6, yet does not alter the localization of its heterodimeric partner, MSH2, suggesting important differences between the human MMR proteins and the lower eukaryotic counterparts. A cancer-derived point mutation in MSH6 (S285I), within the general region of the NLS alters the nuclear localization of the protein, suggesting that not only protein function, but cellular distribution may contribute to carcinogenesis. Given that correct subcellular localization is a prerequisite for all subsequent cellular functions, the identification of unique, cooperative NLSs that provides a new role for an uncharacterized region of human MSH6 is of high significance.

## Results

### Identification of human MSH6 nuclear localization signals

**Figure 2:**
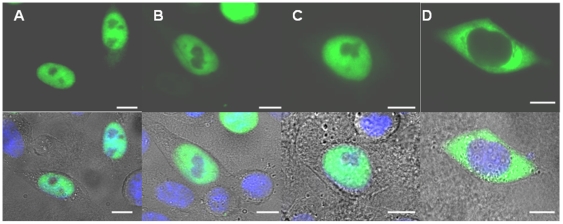
Identification of amino acid stretch in NTR of MSH6 that directs nuclear localization using N-terminal deletion mutants of human GFP-tagged MSH6 in Δ*msh6* cells. Shown are widefield fluorescent images (green) and its overlay with the DIC image to indicate cellular outline. Hoechst staining (blue) indicates the location of the nucleus. All scale bars are 10 µm. (A) GFP-MSH6, full length protein; (B) GFP-MSH6 Δ1–100; (C) GFP-MSH6 Δ1–210; (D) GFP-MSH6 Δ1–399. A clear alteration in nuclear localization is observed upon the truncation of amino acids 211–399.

Maintenance of genomic fidelity requires MSH6, the protein subunit of the MSH2/MSH6 heterodimer responsible for correct mismatch recognition, to correctly localize into the nuclear compartment. Examination of the spatial distribution of *endogenous* MSH6 in HEK 293 and DLD-1 + chr.2 (chromosome 2 complemented colorectal cancer cell line DLD, which is deficient in *hmsh6* [23]) cells reveals that MSH6 is primarily nuclear (Figure S2A, B). This distinct nuclear compartmentalization within the cell is recapitulated by both fluorescently tagged proteins (Figure 2A) and FLAG-tagged constructs (Figure S3). Given that no significant difference in compartmentalization was observed between the fluorescently-tagged proteins and constructs with the shorter FLAG tag (Figure S3), only GFP-tagged constructs will be reported. It is noteworthy that endogenous or tagged MSH2 is distributed between the nucleus and cytoplasm; its localization is independent of MSH6 (Figure S2, 3G; manuscript submitted). This finding and the separate nuclear import mechanism observed in MSH2 will be discussed in more detail elsewhere (manuscript submitted and in preparation).

**Figure 3:**
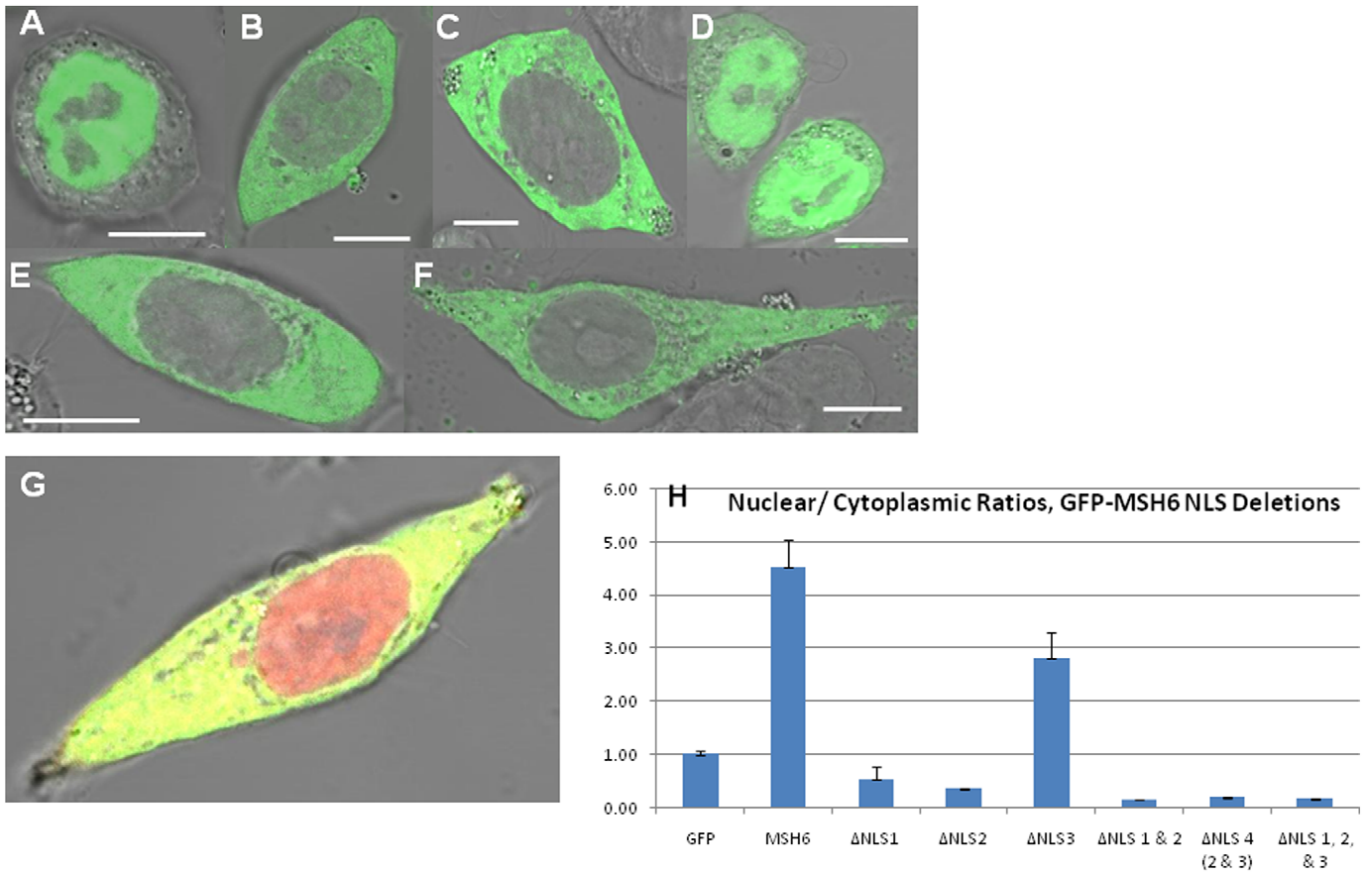
Deletion of predicted NLS from human GFP-tagged MSH6 in Δ*msh6* cells. Shown are the fluorescent view (green) and its overlay with the DIC image to indicate cellular outline. All scale bars are 10 µm. (A) GFP-MSH6, (B) GFP-MSH6 ΔNLS1, (C) GFP-MSH6 ΔNLS2, (D) GFP-MSH6 ΔNLS3, (E) GFP-MSH6 ΔNLS2/3, (F) GFP-MSH6 ΔNLS1/2/3. (G) Co-transfection with dsRed-MSH2 and GFP-MSH6 Δ1–399, (H) Nuclear to cytoplasmic ratios quantified for the corresponding NLS deletion mutants shown.

GFP-MSH6 is predominantly found in the nuclear compartment of the cells (Figure 2A and 3A). Sequential truncations of the NTR of MSH6 reveal a distinct alteration in the localization pattern between residue 211 and 399 of the NTR. As shown in Figure 2B–C, deletion of the first 100 amino acids and 210 amino acids of the NTR, respectively, result in no alteration in nuclear localization; however, deletion of the first 399 amino acids results in complete nuclear exclusion of MSH6 (Figure 2D). These data demonstrate that amino acids 211–399 of the NTR play a role in nuclear localization, and are independent of the PCNA binding domain, which is found in the first 10 amino acids and the PWWP domain contained within the first 210 amino acids (Figure 1A). To determine that the mutant does not affect any essential function of the protein that could be important for its cellular distribution, we determined the ability of the wild type and mutant GFP-tagged proteins to interact with MSH2. Results showed that wild type and mutant MSH6 equally pulled out MSH2 in the IP experiment that was performed with a GFP antibody (Figure S1).

To identify the specific localization signals, we screened the amino acid sequence of MSH6 for putative nuclear localization signals. Using both PSORTII (http://psort.ims.u-tokyo.ac.jp/form2.html) and NLSmapper (http://nls-mapper.iab.keio.ac.jp/cgi-bin/NLS_Mapper_form.cgi [22]), four putative NLSs were identified for MSH6: three monopartite, NLS1 ^246^KKRR^249^, NLS2 ^298^RKRKR^302^, and NLS3 ^311^KRK^313^, and one potential bipartite sequence that combines NLS2 and 3 ^298^RKRKRMVTGNGSLKRK^313^ (Figure 1A,B). This bipartite varies significantly from experimentally determined bipartite NLS sequences, appearing inverted in sequence [20], [22], [24] and is only conserved among MSH6 proteins of higher eukaryotes (Figure 1B).

Deletion of the NTR, which contains the three predicted NLSs, results in complete nuclear exclusion of MSH6 (Figure 2D); therefore, to determine the functionality of each putative NLS sequence, we deleted each sequence individually (Figure 3B–F). The GFP-MSH6 NLS mutants were transiently transfected into *msh6*-deficient DLD-1 cells (which contain endogenous MSH2), which have been described extensively [25], and their subcellular localization examined by confocal microscopy (Figure 3B–F).

Transient transfection results in variable levels of protein expression, which cannot be accurately quantified. To deal with this problem, we examined the individual intensity of cells transiently transfected with GFP-MSH6 and created threshold ranges that grouped cells with similar intensity and produced the correct localization pattern observed for endogenous wild type MSH6. Three threshold ranges were identified: low, medium and high (for settings see Material and Methods). The low and medium thresholds best recapitulate the compartment-specific localization of endogenous MSH6, while the high threshold contained cells with large aggregates of GFP, which created intensely saturated bright spots. Cells at the “high level” were therefore omitted from analysis. The medium threshold was used to obtain high resolution images for these studies since the fluorescence signal showed less photobleaching than the low threshold settings. For each cell, the fluorescence intensities of MSH6 in the nucleus and the cytoplasm were measured and the ratio of nuclear/cytoplasmic (N/C) fluorescence was calculated (Materials and Methods).

As illustrated in Figure 3B–H, deletion of the individual putative NLSs resulted in a distinct pattern of localization. Deletion of monopartite NLS2 (Figure 3C) and bipartite NLS2/3 (Figure 3E) resulted in nuclear exclusion of MSH6 (N/C = 0.35±0.02 and N/C = 0.19±0.01, respectively, Figure 3C, E, H, Table 1). Deletion of monopartite NLS1 also dramatically diminished the nuclear import (N/C = 0.53±0.25, Figure 3B, H, Table 1); however, a mixed localization pattern was observed for this mutant with ∼40% of the cells showing complete nuclear exclusion and the remaining cells showing a varying degree of nuclear localization (data not shown). Deletion of NLS3 results in impairment of nuclear import to a lesser degree than the other NLSs, (N/C = 2.81±0.02, p = 0.0108, Figure 3D, H, Table 1). The *Δmsh6* cells used for transfections in these experiments contain endogenous MSH2, making it unlikely that the lack of protein partner has an effect on the cellular distribution; however, to verify this, we co-transfected wild type dsRed-MSH2 with GFP-MSH6 Δ1–399. As seen in Figure 3G, the localization of dsRed-MSH2 is unaltered and present in the nucleus and cytoplasm.

**Table 1:** Nuclear to Cytoplasmic Ratios (N/C) for GFP-MSH6 NLS deletions and NLS PK-GFP fusions.

GFP-MSH6 NLS mutants	N/C Ratio ± SEM	NLS PK-GFP fusions	N/C Ratio ± SEM
Endogenous MSH6 (DLD1 + chr. 2)	9.03±0.39		
GFP_free_	1.00±0.06		
GFP-MSH6	4.54±0.5	PK-GFP	0.20±0.02
GFP-MSH6 ΔNLS1	0.53± .25	No NLS PK-GFP	0.36±0.06
GFP-MSH6 ΔNLS2	0.35±0.02	NLS1 PK-GFP	1.40±0.16
GFP-MSH6 ΔNLS3	2.81±0.48	NLS2 PK-GFP	1.49±0.1
GFP-MSH6 ΔNLS 1/2	0.15±0.01	NLS3 PK-GFP	0.61±0.05
GFP-MSH6 ΔNLS 2/3	0.19±0.01	NLS1/2 PK-GFP	2.35±0.15
GFP-MSH6 ΔNLS 1/2/3	0.16±0.01	NLS1/3 PK-GFP	2.79±0.21
GFP-MSH6 Δ1-399	0.1±0.01	NLS2/3 PK-GFP	1.52±0.14
GFP-MSH6 S227I	3.63±.45	NLS1/2/3 PK-GFP	3.95±0.28
GFP-MSH6 S285I	1.78±.26		
GFP-MSH6 K295R	4.22±0.7		
GFP-MSH6 S315F	3.95±0.46		

Shown are standard errors for the indicated number of quantified cells.

Forty to sixty cells were quantified for each construct.

### NLSs in MSH6 NTR direct nuclear localization

**Figure 4:**
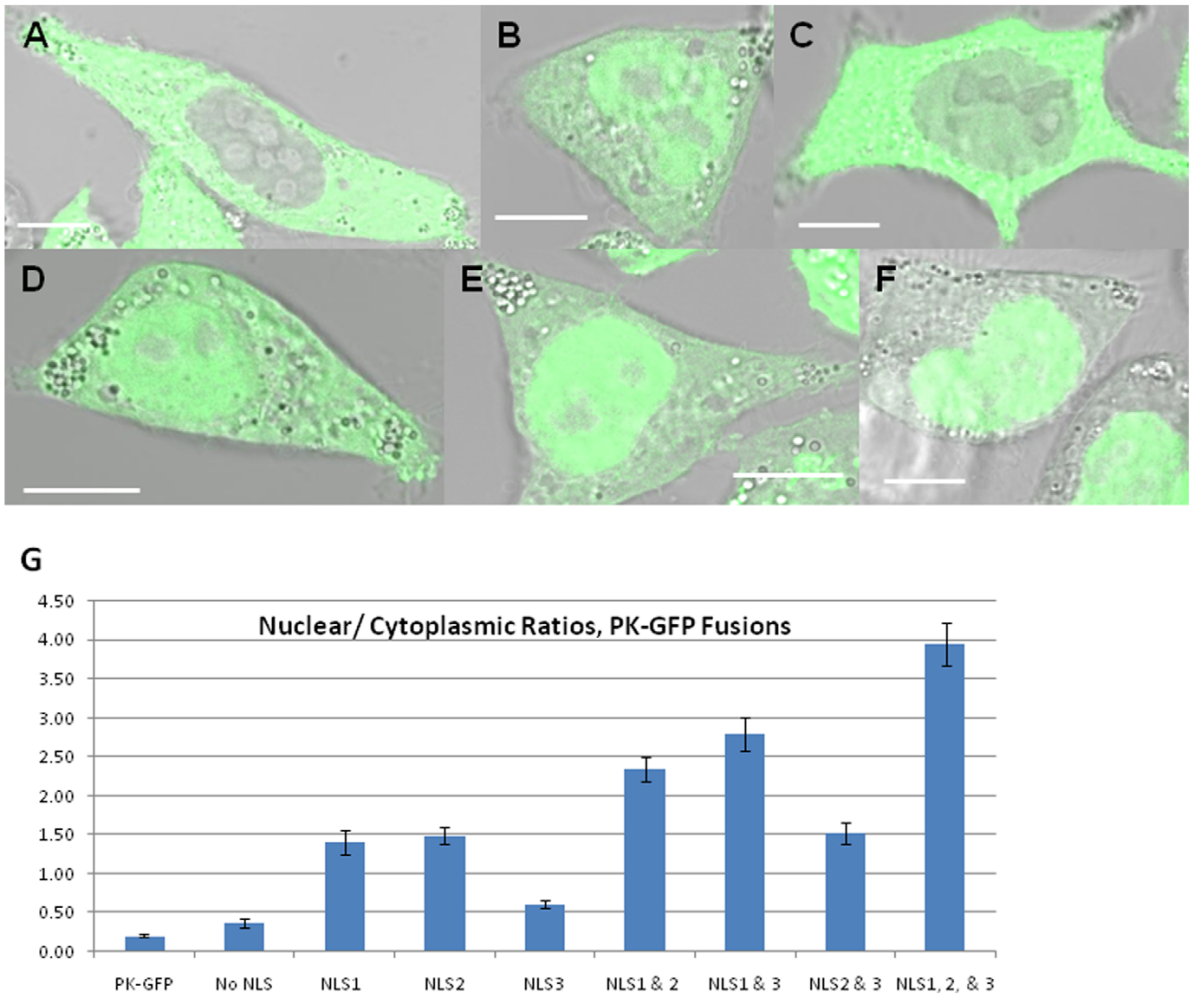
Predicted NLSs are validated by their ability to import a cytoplasmic protein, pyruvate kinase, into the nucleus in Δ*msh6* cells. (A) Pyruvate kinase-GFP fusion (PK-GFP); (B) NLS1 PK-GFP; (C) NLS3 PK-GFP; (D) NLS2/3 PK-GFP; (E) NLS 1/3 PK-GFP; (F) (MSH6 NLS1, 2&3)-PK-GFP. (G) Nuclear to cytoplasmic ratios quantified for NLS pyruvate kinase fusions. The no NLS fusion contains only the intervening amino acids between the predicted nuclear localization sequences to validate that this flanking sequence does not accomplish nuclear import in the absence of the predicted NLS.

To determine whether the identified NLSs are sufficient to mediate nuclear import, we expressed GFP-tagged cytosolic chicken muscle pyruvate kinase (CMPK) [26], (PK-GFP). This protein construct lacks an NLS and shows nuclear exclusion of the fusion protein (N/C = 0.22±0.02) (Figure 4A, Table 1). Given the proximity of the NLSs in the NTR, amino acids 241–315 were fused to the N-terminus of the PK-GFP reporter plasmid (NLS1/2/3 PK-GFP, Figure 4B). Individual NLSs and pair-wise combinations were created by site-directed mutagenesis, and the localization of these proteins was quantified as described above.

NLS1/2/3 PK-GFP showed import levels similar to those observed for wild type GFP-MSH6 (Compare N/C = 3.95±0.28 to N/C = 4.54±0.5, Figs. 3A and 4B and G, Table 1). However, examination of the individual putative NLSs revealed they directed the cytosolic PK-GFP fusion protein into the nucleus to a lesser degree. NLS1, NLS2, and NLS2/3 attained similar levels of localization (N/C of 1.4±0.16, 1.49±0.1, 1.52±0.14, respectively) (Figure 4C, image not shown, E, G, Table 1,), while, NLS3 shows no significant accumulation over the negative control (Figure 4D, G, Table 1). Paired combinations of NLS1 with NLS2 (N/C = 2.35±0.15, image not shown, Figure 4G, Table 1) or NLS3 (N/C = 2.79±0.21, Figure 4F, G), showed significant improvement in nuclear accumulation (p<0.0001), despite the 49 or 62 intervening amino acid between these sites, though still failed to achieve comparable levels to NLS1/2/3 PK-GFP. As a control, a fusion containing only the intervening residues between all three NLSs showed no significant nuclear accumulation (N/C = 0.36±0.06, image not shown, Figure 4G, Table 1), validating import is directed by the identified NLSs.

### Kinetic modeling suggests cooperativity between NLSs

**Figure 5:**
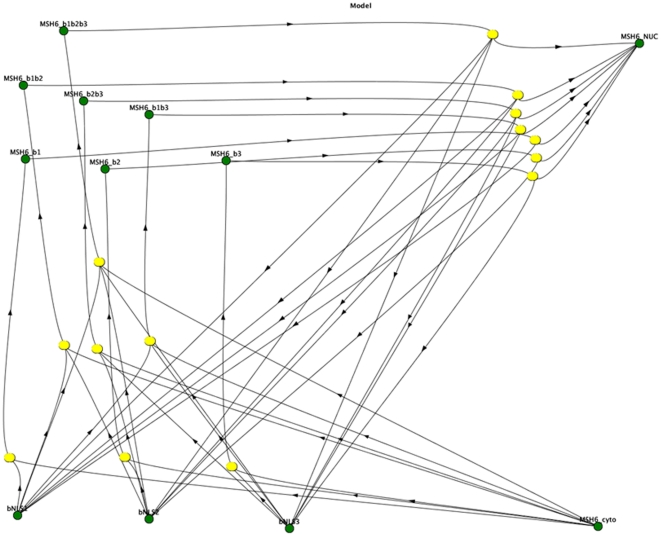
The full kinetic model for import of MSH6 into the nucleus with 12 species and 14 reactions, is shown diagrammatically. Each green ball is a molecular species or complex and each yellow oval is a reaction. The arrows into a yellow oval indicate those species are reactants and errors out indicate the species are products. The different species and reaction types are organized spatially for clarity. Each of the four base species, MSH6, and the binders to NLS1, NLS2, and NLS3 (bNLS1, bNLS2, bNLS3, respectively, are shown at the bottom as green circles as species that are reactants for the formation reactions to which they are linked by inward arrows. The formation reactions that lead to complex formation are shown in yellow in the middle, with the complexes formed shown at top as green circles; the complexes formed are linked to the correct formation reactions by outward arrows. The complexes are named appropriately, so that for example, MSH6_b1 is the complex of MSH6 with the binder to NLS1 and is connected via a yellow reaction oval to bNLS1 (the binder to NLS1) and MSH6 in the cytoplasm. The disintegration reactions that implicitly model transport into the nucleus through complex disintegration are shown at top right in yellow, where they connect the appropriate complex to the correct molecular species, leading to MSH6 in the nucleus. For example, MSH6_b1 is connected to MSH6 in the nucleus and bNLS1 (the binder to NLS1) via a disintegration reaction.

To validate cooperation between the identified NLSs, we used the experimental data to create a kinetic model of the import of cytoplasmic protein into the nucleus. The reaction scheme used is a simple implicit model of complex formation and transport (see Figure 5 for the full model, presented diagrammatically with 12 species and 14 reactions). This model addresses the question of whether or not the changes in nuclear transport can be explained by the existence of multiple, independent NLSs, assuming transferability between mutant experiments, or on the requirement for cooperativity.

The key parameters in the model are the relative stabilities of each complex in the nucleus relative to their stability in the cytoplasm. Based on these parameters, the model can estimate relative changes in free energies by determining how the relative stabilities change as the models becomes increasingly complex (Table 2). The first model is a single NLS model (Figure 5, MSH6_b1, _b2 or _b3, referring to MSH6 bound to an import protein binding to NLS1 or NLS2 or NLS3 separately); kinetic parameters can be scanned to determine those that are most consistent with the experimental data. The next model contains two NLSs. In a non-cooperative model, the binding to both NLS 2 and NLS 3 would be the same as the sum of the binding the NLS2/3. This result is not observed, instead a change in stability is required when both NLS 2/3 are bound; this is a thermodynamic signature of cooperativity. The same observation is made for NLS 1/2, NLS 1/3 and for the binding of all three NLSs.

**Table 2:** Cooperativity estimates from kinetic model.

NLS	Cooperativity (Kcal/mol)
1/2	2.64
1/3	2.94
2/3	1.71
1/2/3	2.58

The cooperativity estimates are obtained by converting rate constants into free energies and then subtracting from the free energy of binding of the pairs the sum of the individual free energies. For the triplet, the cooperativity is estimated by subtracting a free energy for a pair and the remaining single free energy, and using the minimum of the three values found.

### Heterodimerization is not a prerequisite for nuclear import in undamaged cells

**Figure 6:**
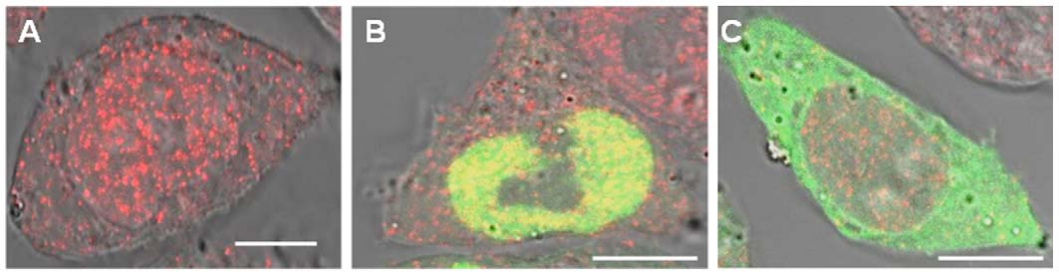
A deficiency in MSH6 does not alter the localization of MSH2. Shown is the localization of endogenous MSH2 in Δ*msh6* cells and in Δ*msh6* cell transfected with GFP-MSH6. Scale bars are 10 µm. (A) Alexa546 MSH2, (B) Alexa546 MSH2 and GFP-MSH6, (C) Alexa546 MSH2 and GFP-MSH6 ΔNLS 1/2/3.

Our initial data suggested localization of individual proteins independent of their heterodimeric partner. To determine if the localization of human MSH6 indeed did not direct the localization of MSH2, as reported for the yeast orthologs, we utilized immunofluorescence to visualize the localization of endogenous MSH2 in *msh6* deficient cells. The genetic deficiency would result in mislocalization of MSH2, if MSH6 was required for its nuclear import. In contrast to the yeast proteins, lack of MSH6 did not affect or alter the cellular distribution of MSH2 (Figure 6A), which is also evident by co-transfection of the deficient MSH6 with wild type MSH2 (Figure 3G). When MSH6 expression is restored by transient expression of our GFP fusions, no alteration in MSH2 cellular distribution is observed (Figure 6B,C).

These data are in striking difference to previous results in yeast or after exposure to DNA damaging agents, and suggest that the presence of human MSH6 is not a pre-requisite for nuclear import of MSH2 in undamaged cells. To rule out that endogenous MSH3 takes over import of MSH2, we repeated experiments in cells lacking both endogenous MSH3 and MSH6 (Figure S5). The localization of endogenous MSH2 in cells lacking both MSH3 and MSH6 was unaltered and remained distributed between the nucleus and cytoplasm (Figure S5A). Expression of GFP-MSH6 ΔNLS2/3 did not alter the localization of endogenous MSH2 in these cells (Figure S5B).

### Cancer-derived point mutation in MSH6 alters subcellular localization

A number of cancer-derived point mutations have been identified near or within the NTR region (a.a. 241–315), though none of these mutations directly affect the MSH6 NLS identified herein. To determine if these point mutations created defects in the subcellular localization of MSH6, which would be missed by *in vitro* biochemical analyses, we introduced several of these point mutations into the GFP-MSH6 fusion and quantified the nuclear to cytoplasmic ratios. While most point mutations showed no significant alteration in cellular localization, one, S285I (Figure 1), showed a significant defect in nuclear localization (p<0.0001; Table 1). This mutation was previously characterized by a low microsatellite instability phenotype [27], and a lack of functional defects, such as defective heterodimerization or ATP hydrolysis [28]. Its altered cellular localization is likely due to changes in the overall conformation of the NTR regions rather than a direct effect on the nuclear localization sequences; but it demonstrate that a single point mutation can affect cellular localization and may even contribute to carcinogenesis, without affecting protein function.

## Discussion

These studies for the first time identified a novel mechanism for nuclear import of human MSH6 that differs from that identified in lower eukaryotic cells in that it is independent of its heterodimeric partner.

Correct localization of MMR proteins into the nucleus is critical to their proper function. Two classes of NLSs have previously been identified, monopartite and bipartite, and loose consensus sequences established for both. Examination of human MSH6 revealed three putative monopartite NLSs (NLS1, 2 and 3) and one potential bipartite NLS, a combination of two identified monopartite sequences (NLS2/3), in the NTR of MSH6, a substantial N-terminal protein region that has evaded functional characterization to-date. Inspection of the NTR region of other higher eukaryotes shows similar and closely spaced NLSs (Figure 1B), suggesting that cooperative NLSs may be utilized by other higher eukaryotes to localize MSH6 into the nuclear compartment. In contrast, the yeast Msh6 NTR does not contain a similar region of clustered NLSs, indicating evolutionary separation between lower and higher eukaryotes.

The monopartite NLS1 and NLS2 are high scoring NLSs in agreement with experimentally determined localization sequences, while NLS3 is a low scoring, non-classical NLS [22]. The general sequence containing these short 2–3 basic residues has been experimentally verified to promote nuclear import [29], [30]. The predicted bipartite sequence containing NLS2/3 is also a high scoring NLS predicted to have significant levels of nuclear import; however the consensus sequence appears inverted from experimentally verified bipartite sequences [22]. Experimental validation of these putative NLSs showed dramatic decreases in nuclear transport when NLS1, NLS2, NLS 2/3, or NLS 1/2/3 were deleted (Figure 3G), while NLS3 only produced a slight defect in nuclear import when deleted (Figure 3G).

To confirm that these putative NLSs are sufficient to mediate nuclear import, NTR region 241–315 was fused to a cytoplasmic pyruvate kinase-GFP fusion and the individual NLSs, as well as combinations of individual NLSs were examined. Nuclear accumulation was observed for NLS1, NLS2, and NLS2/3 (Figure 4G), and weak accumulation for NLS3 alone (Figure 4G), consistent with the predictions for the consensus matching of these individual predicted NLSs [22]. However, the ability of NLS2 alone to direct nuclear import to a similar degree as NLS2 and 3 together is inconsistent with NLS2 and 3 representing a true bipartite sequence, as described for other proteins [24], [31]. Bipartite nuclear localization signals are interdependent basic domains, where the large monopartite-like domain is insufficient to direct nuclear import of a cytoplasmic protein in the absence of its partner domain [20], [24]. Our data suggest that NLS2 and NLS3 together do not form a bipartite nuclear localization signal.

The individual NLSs were not capable of directing nuclear protein import to the same degree as the combinations of two or all three of the NLSs or to the same degree as wild type MSH6 (Figure 3H and 4G). Since levels of NLS activity vary dependent on the sequences flanking the NLS [22], the proximity of the predicted NLSs in the NTR made a cooperative effect between the individual NLSs an intriguing possibility. Cooperative nuclear localization sequences have been previously described for a number of proteins, whether it be between different NLSs sequences [32], [33], [34], [35] or between NLSs and other protein domains [36], [37], [38]. Since amino acids 241–315, containing all three NLSs, significantly increases nuclear import over the effects of the individual or pair-wise NLSs (Figure 4G), cooperation between the sequences was considered. While the cooperative effect of other proteins has been previously described, the underlying mechanism by which these protein domains cooperate is unknown.

To support our proposed cooperativity, we utilized kinetic modeling. This method used the current experimental data to model the full kinetic process of nuclear import and to estimate missing parameters, such as the stability of the various NLS/MSH6 complexes (Figure 5 and description therein). The kinetic model supports cooperativity between the three individual monopartite NLSs to efficiently translocate MSH6 into the nuclear compartment.

The cooperative action of the tripartite NLS ensures that human MSH6 is efficiently localized into the nucleus. While NLS1 and NLS2 appear to be the more dominant regions, NLS3 significantly enhances the efficacy of nuclear import by NLS1 alone or in combination with NLS2. These hierarchical NLSs suggest the evolution of redundant, though less effective, delivery systems for ensuring human MSH6 is localized into the nuclear compartment that are distinct from lower eukaryotic systems.

In contrast to yeast, loss of nuclear localization in human MSH6 has no discernable effect on its heterodimeric partner, MSH2; suggesting that MSH6 does not direct the localization of MSH2 (Figure 3G, 6). Localization of yeast proteins to the nuclear compartment was found to be dependent on a reciprocal regulation between Msh2 and Msh6 [13]. Yeast Msh2 contains the necessary NLSs to direct itself independently to the nucleus; however, the proteins levels of Msh2 were found to be diminished in the absence of Msh6 [13]. Yeast Msh6 contains no putative NLSs and is dependent on Msh2 for transport into the nucleus [13]. Taken together, these results suggested that heterodimer formation was required to efficiently localize the yeast Msh2 and Msh6 into the nucleus and stabilize the monomeric proteins [13].

In striking contrast, human MSH2 contains no putative NLSs, while MSH6 contains three cooperative NLSs, and thereby differs from the yeast proteins. Despite the lack of NLSs, the absence of human MSH6 does not abrogate localization of human MSH2 into the nucleus, nor does it diminish the protein levels of MSH2 (Figure S4). This indicates that MSH2 and MSH6 can localize independently into the nucleus and that heterodimerization of MSH2 and MSH6 is not a pre-requisite for the nuclear import of the human proteins, in significant contrast to the yeast proteins.

Previous studies with human MSH2 and MSH6 suggested that heterodimerization was a pre-requisite for nuclear import in the presence of DNA damage [16], [17]. Protein levels of MSH2 and MSH6 were found to increase in the nucleus after DNA damage, and nuclear transport of MSH2 was abrogated in MSH6 deficient DLD-1 cells [16]. However, as shown in Figure 6, human MSH2 clearly localizes into the nuclear compartment independent of human MSH6 in DLD-1 cells. Further, a recent study examined the accumulation of human MMR proteins at laser-irradiated sites and found that MSH6 accumulates at laser-irradiated sites in the absence of MSH2 [19], suggesting human MSH2/6 heterodimer formation is not required in the response to DNA damage. This does not eliminate the possibility that MSH2 heterodimerizes with any of its numerous other binding partners to accomplish nuclear import, but does demonstrate that MSH2 and MSH6 achieve nuclear localization independently and opens new avenues of investigation into their independent regulation. To determine if heterodimerization with MSH3, its alternate partner, affects cellular localization of MSH2, we performed experiments in cells that are deficient in both MSH3 and MSH6. Our data demonstrate that the absence of MSH3 does not alter the localization of MSH2 (Figure S5).

Mutations in human MSH6 are present in 12% of patients with hereditary non-polyposis colorectal cancer [39]. Interestingly, 26 out of the 91 point mutations identified in MSH6 are located to the NTR [39]. Mutations in MSH6 are associated with later age onset than mutations in MSH2, and mutations are found more often in endometrial cancer [40], [41]. Recently a link between mutations in MSH6 and childhood leukemia/lymphoma was identified [42]. While a majority of MSH6 mutations are truncations or deletions, about 30% of the MSH6 variants reported are single amino acid substitutions [39]. A number of studies have attempted to determine the biochemical defects of these mutations in MSH6 [28], [43]; however, the functional consequences in whole cells are largely unknown.

The fact that defects in MMR proteins may alter the subcellular localization without directly affecting protein function has been largely ignored up to this point. A number of cancer-derived mutations in MSH2 and MLH1 have been identified that alter subcellular localization [21], [44], [45]. In the region identified to be critical for nuclear localization of MSH6, we identified a single point mutation, S285I, which significantly alters the subcellular localization of MSH6. This mutation is associated with a low microsatellite instability phenotype [27]; however, *in vitro* analysis of this protein found no defects in its ability to heterodimerize with MSH2 or hydrolyze ATP [28], despite its association with cancer. Though this mutation does not reside directly in any of the identified NLS, it has previously been shown that levels of NLS activity are dependent on the sequences flanking the NLS [22]. The location of this mutation in the region flanking the two dominant nuclear localization sequences, NLS1 and 2, makes it probable that this cancer mutation disrupts some aspect of the cooperation between NLSs, reducing the nuclear accumulation to levels consistent with a single NLS. Alternatively, the mutation can simply cause unfavorable structural changes in the region. These results simply demonstrate that a single point mutation in the vicinity of the NLS can affect cellular localization of proteins. This is the first example of an MSH6 cancer mutation to show consequences for protein localization rather than function, an unexplored source of promoting carcinogenesis. These data caution on the importance of whole cell behavior when studying cancer-associated mutations and their effects.

## Materials and Methods

### Plasmids

GFP-MSH6 was a kind gift from A. Yasui and was described previously [19]. Site-directed mutagenesis was utilized to create the GFP-MSH6 mutations described. The GFP-MSH6 truncations were created by PCR amplification of amino acid residues, 101-1360, 211-1360 or 400–1360 of MSH6 by primers containing *XhoI* and *NotI* restriction sites. The PCR products were then digested and ligated into the modified pEGFP-C1 vector provided by Yasui [19]. GFP-MSH6 and GFP-MSH6 Δ1–399 functionality was determined by interaction with MSH2 (Figure S1). Additionally, a wild-type MSH6 and MSH6 Δ NLS 2/3 with a FLAG epitope (DYKDDDDK) in place of the GFP on the N-terminus were created by PCR amplification. All clones were sequence verified.

Cytoplasmic pyruvate kinase (p3PK) was a kind gift of J. Frangioni and was previously described [26]. The pyruvate kinase (PK) cassette was removed from the p3PK vector by digestion with *HindIII* and *BamHI*, and ligated into pEGFP-N2. The frameshift between the PK and the GFP was corrected by site-directed mutagenesis, creating PK-GFP. Amino acids 241–315 of MSH6 were PCR amplified with primers containing *NheI* or *BamHI*. The PCR product was digested and ligated into the *NheI* and *BglII* sites of PK-GFP, creating NLS1,2,&3 PK-GFP. Site-directed mutagenesis was used to remove individual NLSs, creating the NLS variants used. All clones were sequence verified.

Colorectal cancer cell line DLD-1, deficient in *msh6,* and its complemented, *msh6* proficient counterpart, DLD-1 + chr.2 have been extensively described [23], [25] These cells were cultured under standard culture conditions, using DMEM-F12 media (Gibco) with 10% FBS. All cell lines were maintained at 37°C in a 5% CO_2_ atmosphere. Cells were transiently transfected using Lipofectamine 2000 (Invitrogen).

### Immunoprecipitation and western blotting

Whole cell lysates were prepared by resuspending cell pellets in NP40 lysis buffer, then sonicated to lyse cells. 500 µg of the lysate was added to Dynabeads Protein G (Dynal, Invitrogen) pre-bound with either anti-GFP antibody (abcam ab290). Anti-GFP beads were incubated for 40 minutes at room temperature, then washed and eluted according manufacturer's protocol.

Eluted complexes were analyzed by SDS-Page. The presence of protein was detected by 1 µg/mL anti-MSH2 (BD Biosciences 556349) and 1 µg/mL anti-MSH6 (BD Biosciences 610918) against either mismatch repair protein.

### Immunofluorescence

Cells were grown on poly-lysine coated dishes (MatTek Corp) and fixed with a 10% neutral buffered formalin solution (Thermo Scientific). After incubation in blocking solution, slides were incubated with 2 µg/mL anti-MSH2, (Santa Cruz sc-494) or 4 µg/mL anti-FLAG M2, (Sigma F1804). Fluorescently tagged secondary antibodies were used to visualize the protein and its location. Anti-MSH2 antibody was recognized by an Alexa Fluor 546 goat anti-rabbit antibody (1∶2500, Invitrogen). Anti-FLAG antibody was recognized by an Alexa Fluor 488 goat anti-mouse antibody (1∶2500, Invitrogen) As controls, secondary antibody alone or cells lacking the epitope for the primary antibody were used.

### Widefield Microscopy

Cultured cells were fixed and labeled as described. Cells were then imaged with a 63x 1.4NA oil immersion objective on a Zeiss AxioObserver widefield microscope (Carl Zeiss MicroImaging, Thornwood, NY) equipped with a Hamamatsu Orca C4742-80-12AG monochromatic digital camera (Hamamatsu, Bridgewater, NJ) and an X-Cite metal halide fluorescent light source with the aperture fully open (Exfo, Mississauga, Ontario). Hoechst stained nuclei were visualized using a UV filter cube (DAPI-1160A, Semrock, Rochester, NY) while GFP-MSH6 was visualized using a GFP filter cube (Filter Set 13, Carl Zeiss MicroImaging, Thornwood, NY). Images were acquired sequentially for each channel using the Volocity Acquisition module (Perkin-Elmer, Waltham, MA). Exposure time, gain, and offset were held constant for all image acquisition.

### Confocal microscopy

Fixed and labeled cells were also imaged using a Zeiss LSM 710 single-photon confocal microscope equipped with the quasar 34-channel spectral detector (Carl Zeiss MicroImaging, Thornwood, NY). 2-D images were acquired using a 63x 1.4NA oil-immersion objective. For GFP imaging, the 488 nm laser line with a 488 long pass dichroic with an emission range of 492–630 nm was used. Hoechst staining could not be visualized on this microscope; therefore a matching DIC image was acquired simultaneously to confirm the position of the nucleus and to select the best focal plane for nuclear imaging. Images were acquired with a pinhole of 1.36 airy unit (AU), a zoom of 1.0, using the 63x, 1.4 NA objective. This resulted in images with individual pixel diameters of 0.09 µm, which is necessary to satisfy the Nyquist sampling requirement for this configuration. Laser power was set at 6% with a constant gain setting of 780, for all quantification acquisitions. Gain setting was determined by examining transiently transfected cells and creating threshold ranges that grouped cells with similar intensity. Two of these groupings, medium (gain 780) and low (gain 1080), best recapitulate the nuclear localization of endogenous MSH6 without aggregation of the GFP protein. The medium threshold was used to obtain high resolution images for these studies, and only cells showing under-saturation with these conditions were imaged and quantified as described below. These same conditions were utilized for quantifying immunofluorescent staining of endogenous MSH6 and FLAG-tagged constructs.

For GFP and Alexa 546 co-localization imaging, a multi-track configuration was used to ensure no excitation cross-talk or emission bleed-through between channels. The 488 nm laser line was used at 6% of maximum intensity and the 543 nm laser line with an emission range of 547–703 nm was used at 30% of maximum intensity. Images were acquired with a pinhole of 1 airy unit (AU), a zoom of 1.5, using the 63x, 1.4 NA objective. This resulted in images with individual pixel diameters of 0.09 µm, which is necessary to satisfy the Nyquist sampling requirement for this configuration. Zen 2009 software was used for all image acquisition.

### Fluorescence Intensity Analysis

The intensity of the fusion protein fluorescent signal in the nucleus and cytoplasm was compared quantitatively with acquisition settings described above. Images were then analyzed using the Volocity Quantitation module (Perkin-Elmer, Waltham, MA). The fluorescent signal from the nuclei of labeled cells was selected by drawing a region of interest (ROI) around each nucleus. A simultaneously acquired DIC image was used to verify the location of the nucleus. The summed signal intensity from all the pixels in the ROI (I_ROI_) was then normalized by the number of pixels in the ROI (N_ROI_) and the background intensity of the image using the following formula: F_obj_ = I_ROI_ – N_ROI_* (I_bkgd_/N_bkgd_). Background was determined by drawing an ROI in an empty region of the image and determining the summed signal normalized to the number of pixels (average intensity)[46]. The fluorescent signal of the cytoplasm was determined by drawing an ROI around the entire cell and following the same procedure as described above to determine the fluorescent signal normalized to background. The contributions of the nucleus were then subtracted from this value to give the fluorescent intensity of the cytoplasm alone. This normalized intensity analysis was used to compare the distribution of GFP-fusion proteins within the nucleus and cytoplasm of individual cells. Forty to sixty cells were analyzed in this manner for each mutant.

Accumulation of free GFP in the nucleus of DLD-1 cells was measured as a control, and a homogeneous fluorescent signal was noted in both nuclear and cytoplasmic compartment (N/C = 1.1±0.06). All N/C ratios were normalized to the free GFP measurement.

### Computational Cell Model

The computational cell model was constructed using the Virtual Cell modeler from the National Resource for Cell Analysis and Modeling (http://www.nrcam.uchc.edu/index.html)[47], [48]. In our model, the reactions are solved numerically with mass-action kinetics[47]. Twelve species are included in the full model (MSH6_cyto, MSH6_nuc, the binder to NLS1 (bNLS1), the binder to NLS2 (bNLS2), the binder to NLS3 (bNLS3, MSH6/bNLS1, MSH6/bNLS2, MSH6/bNLS3, MSH6/bNLS1/bNLS2, MSH6/bNLS1/bNLS3, MSH6/bNLS2/bNLS3, MSH6/bNLS1/bNLS2/bNLS3), with intermediate models consisting of only the relevant species. On the time-scales considered here, only two parameters were found to affect the results, not surprisingly: the ratio of the equilibrium constants for dissociation/formation in the nucleus to the cytoplasm, and the concentrations of the species. So the kinetic parameters were fixed save for the dissociation constants within the nucleus. These concentrations and equilibrium constants were varied logarithmically over three orders of magnitude from 1 µM and 1 µM/s, respectively. Final parameters were extracted based on comparison to the experimental data on the import of cyptoplasmic protein: the nuclear/cytoplasmic ratio was matched under the assumption that the complexes are uncommon (<10% of the MSH6 is in complex). The equilibrium constants were then converted into free energies using dG = -RTlnK.

## Supporting Information

Figure S1 **Western blot of MSH2 and MSH6 immunoprecipitated from transiently transfected** ***msh6-*** **deficient DLD-1 cells.** Both GFP-MSH6 and GFP-MSH6 Δ1-399 co-precipitate MSH2 from cell lysates, demonstrating that there is no defect in heterodimerization.
(TIF)

Figure S2 **Immunofluorescence of endogenous MSH2 (Alexa-488, green) and MSH6 (Alexa-647, red) in non-carcinogenic HEK293 cells.** Scale bar is 10 µm.
(TIF)

Figure S3 **Localization of MSH6 tagged with the much smaller FLAG tag, as a control that measured effects are not artifacts of tagging with GFP.** Localization of A. wt MSH6 and B. delta NLS2/3 MSH6 are shown. Nuclear/cytoplasmic ratios for each measured construct are shown in C.
(TIF)

Figure S4 **Western blot of MSH2 and MSH6 protein levels in** ***msh6-*** **deficient DLD-1 cells compared to levels found in non-carcinogenic HEK293 cells.** Whole cell, cytoplasmic and nuclear extracts were probed. Actin was used as a loading control.
(TIF)

Figure S5 **Localization of MSH6 and MSH2 in cells lacking endogenous MSH3 and MSH6.** A: Localization of endogenous MSH2 in cells lacking MSH3 and MSH6. B: Localization of GFP-MSH6-delta NLS2/3 and endogenous MSH2 in these cells.
(TIF)
