# Supplementary figures and images for: Cooperative Nuclear Localization Sequences Lend a Novel Role to the N-Terminal Region of MSH6
Source: PLoS One. 2011 Mar 17;6(3):e17907. doi: 10.1371/journal.pone.0017907 (PMC3060103; doi:10.1371/journal.pone.0017907)

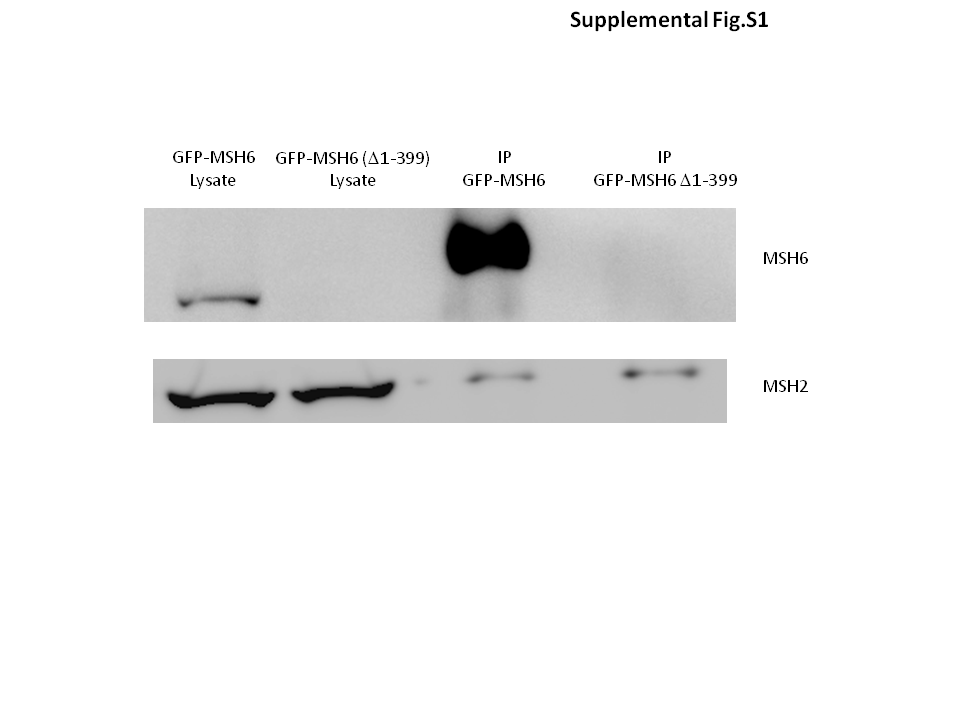

Supplement: Figure S1 — Western blot of MSH2 and MSH6 immunoprecipitated from transiently transfected msh6- deficient DLD-1 cells. Both GFP-MSH6 and GFP-MSH6 Δ1-399 co-precipitate MSH2 from cell lysates, demonstrating that there is no defect in heterodimerization. (TIF) [file pone.0017907.s001.tif]

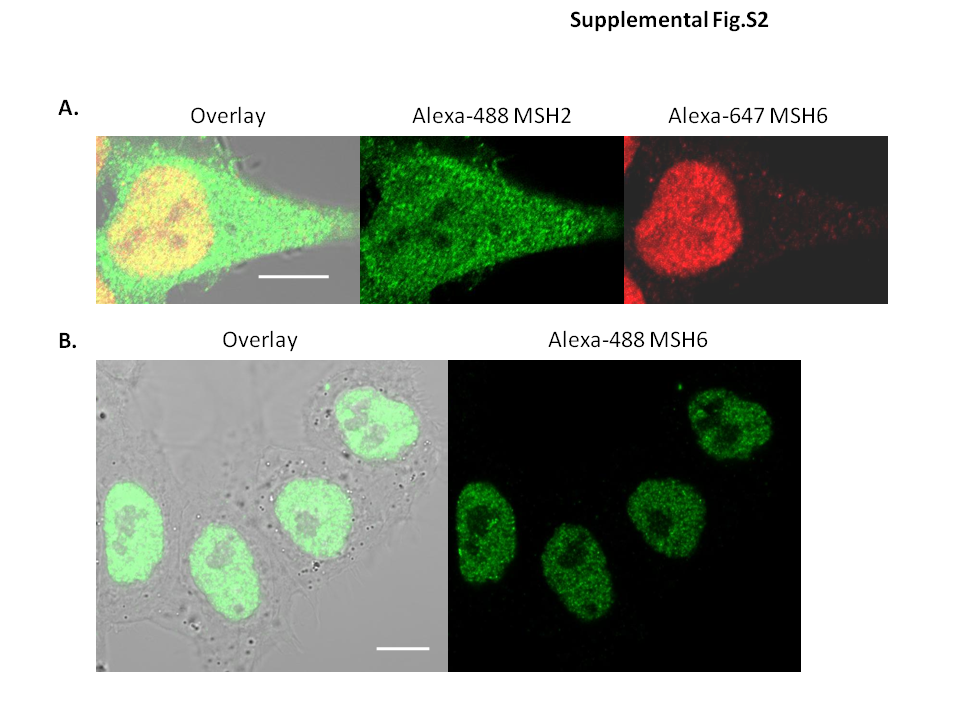

Supplement: Figure S2 — Immunofluorescence of endogenous MSH2 (Alexa-488, green) and MSH6 (Alexa-647, red) in non-carcinogenic HEK293 cells. Scale bar is 10 µm. (TIF) [file pone.0017907.s002.tif]

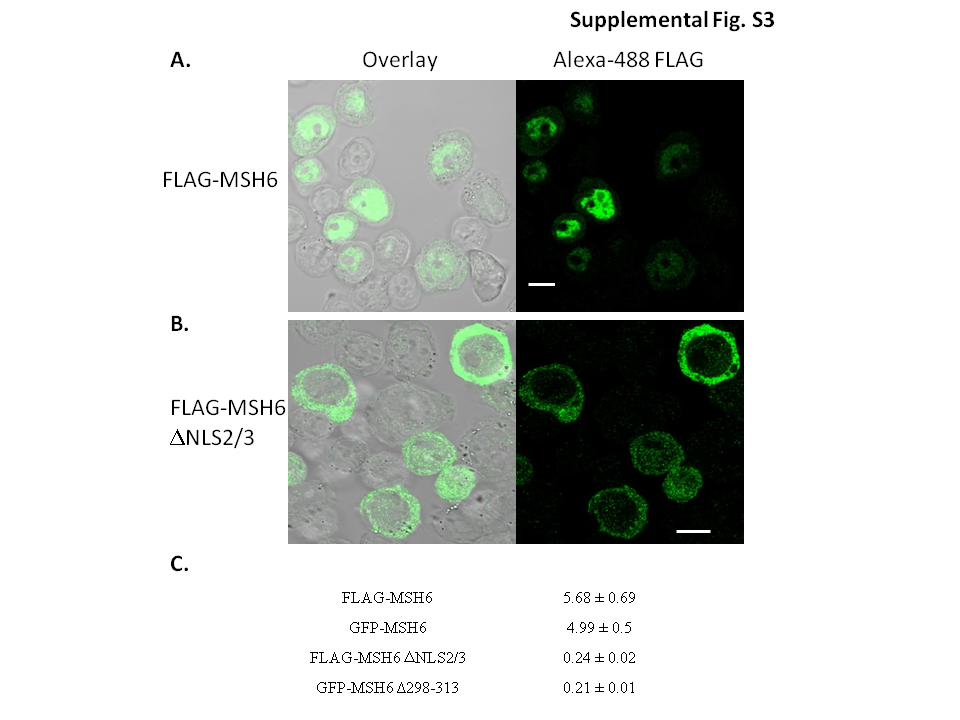

Supplement: Figure S3 — Localization of MSH6 tagged with the much smaller FLAG tag, as a control that measured effects are not artifacts of tagging with GFP. Localization of A. wt MSH6 and B. delta NLS2/3 MSH6 are shown. Nuclear/cytoplasmic ratios for each measured construct are shown in C. (TIF) [file pone.0017907.s003.tif]

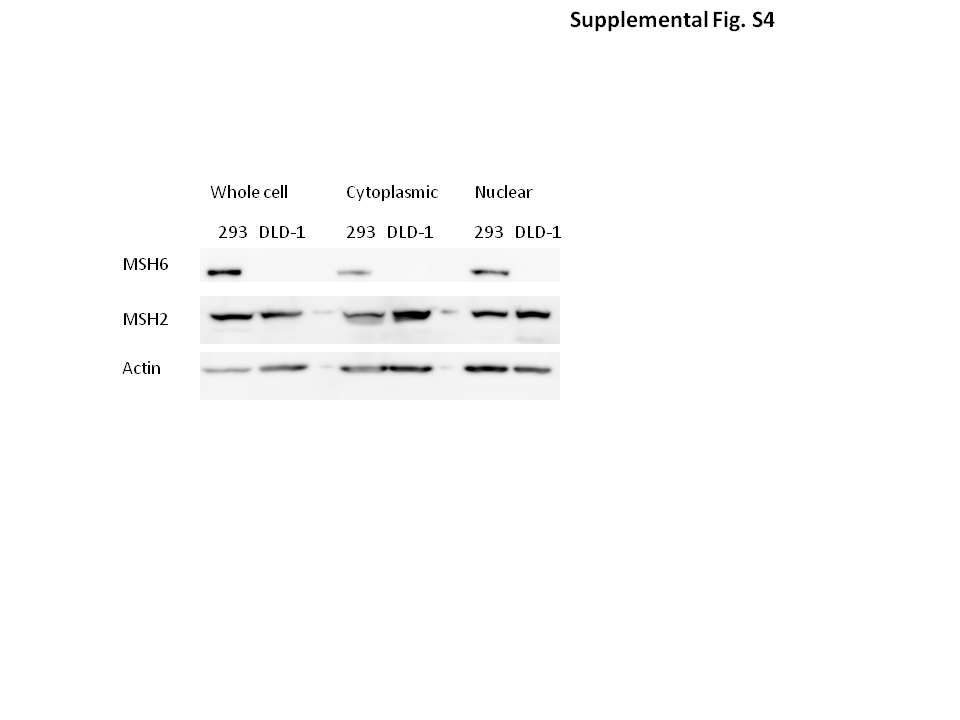

Supplement: Figure S4 — Western blot of MSH2 and MSH6 protein levels in msh6- deficient DLD-1 cells compared to levels found in non-carcinogenic HEK293 cells. Whole cell, cytoplasmic and nuclear extracts were probed. Actin was used as a loading control. (TIF) [file pone.0017907.s004.tif]

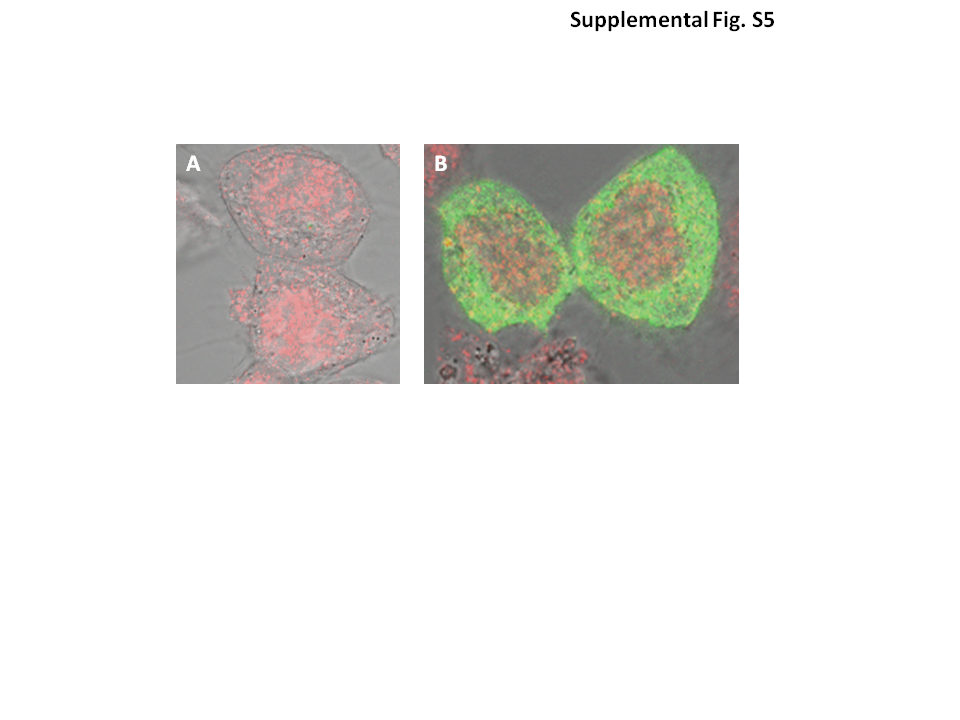

Supplement: Figure S5 — Localization of MSH6 and MSH2 in cells lacking endogenous MSH3 and MSH6. A: Localization of endogenous MSH2 in cells lacking MSH3 and MSH6. B: Localization of GFP-MSH6-delta NLS2/3 and endogenous MSH2 in these cells. (TIF) [file pone.0017907.s005.tif]
